# Supplementary material for: Risk Factors for Hospital Readmission for Clostridioides difficile Infection: A Statewide Retrospective Cohort Study
Source: Pathogens. 2022 May 8;11(5):555. doi: 10.3390/pathogens11050555 (PMC9147200; doi:10.3390/pathogens11050555)
Supplement: Supplementary file 1 [file pathogens-11-00555-s001.zip › pathogens-1673823-Supplementary Material.pdf]

Table S1: Characteristics of CDI cases among patients readmitted with a PCR test

	Index Cases N (%)	Readmitted for CDI N (%)	Not Readmitted for CDI N (%)	*p*-value
Overall	1,227	114	1,113	
**Age**				0.472
18 – 44	180 (15)	18 (16)	162 (15)	
45 – 64	365 (30)	38 (33)	327 (29)	
65 – 79	381 (31)	28 (25)	353 (32)	
Over 80	301 (24)	30 (26)	271 (24)	
**Sex**				0.174
Female	698 (57)	58 (51)	640 (58)	
Male	529 (43)	56 (49)	473 (42)	
**Race/Ethnicity**				0.358
Non-Hispanic White	999 (81)	86 (75)	913 (82)	
Hispanic or Latino	115 (9)	15 (13)	100 (9)	
Non-Hispanic Black	82 (7)	9 (8)	73 (6)	
Other	31 (3)	4 (4)	27 (3)	
**CDI Index Case Classification**				0.373
Community Associated	489 (40)	41 (36)	448 (40)	
Healthcare Associated	738 (60)	73 (64)	665 (60)	
**CDI Treatment Regimen**				0.190
Vancomycin	499 (41)	53 (46)	446 (40)	
Metronidazole	308 (25)	22 (19)	286 (26)	
Sequential	316 (26)	33 (26)	283 (25)	
Concurrent	104 (8)	6 (5)	98 (9)	
**Discharge Disposition**				0.054
Healthcare Facilities	502 (41)	37 (32)	465 (42)	
Home	725 (59)	77 (68)	648 (58)	
**High-Risk Antibiotic°**				0.530
No	442 (36)	38 (33)	404 (36)	
Yes	785 (64)	76 (67)	709 (64)	
**Elixhauser Score**				0.001
Median [IQR]	5 [0 – 11]	6 [0 – 13]	5 [0 – 10]	
**Length of Index Stay (days)**				0.223
Median [IQR]	7 [4 – 13]	6 [4 – 11]	7 [4 – 13]	

Table S2: Adjusted associations of factors with CDI-related readmission among patients readmitted with a PCR test

	Adjusted Odds Ratio	95% CI	95% BCa-CI
**Age**			
18 – 44	Reference		
45 – 64	0.96	0.51 – 1.78	0.51 – 1.88
65 – 79	0.63	0.32 – 1.23	0.29 – 1.26
Over 80	1.00	0.50 – 2.00	0.46 – 2.19
**Sex**			
Female	Reference		
Male	1.21	0.81 – 1.81	0.78 – 1.90
**Race/Ethnicity**			
Non-Hispanic White	Reference		
Hispanic or Latino	1.31	0.70 – 2.43	0.64 – 2.42
Non-Hispanic Black	1.23	0.57 – 2.65	0.55 – 2.54
Other	1.70	0.56 – 5.13	0.47– 4.81
**CDI Index Case Classification**			
Community Associated	Reference		
Healthcare Associated	1.41	0.88 – 2.25	0.87 – 2.25
**CDI Treatment Regimen**			
Vancomycin	Reference		
Metronidazole	0.62	0.36 – 1.05	0.35 – 1.03
Sequential	1.06	0.66 – 1.73	0.62 – 1.73
Concurrent	0.47	0.19 – 1.17	0.17 – 1.35
**Discharge Disposition**			
Healthcare Facilities	Reference		
Home	1.70	1.06 – 2.74	1.04 – 2.88
**Fluoroquinolones**			
No	Reference		
Yes	1.59	1.02 – 2.48	1.01 – 2.41
**1st/2nd Cephalosporins**			
No	Reference		
Yes	0.83	0.47 – 1.46	0.46 – 1.46
**3rd/4th/5th Cephalosporins**			
No	Reference		
Yes	0.98	0.63 – 1.54	0.62 – 1.60
**Clindamycin**			
No	Reference		
Yes	0.99	0.40 – 1.98	0.36 – 2.40
**Penicillin**			
No	Reference		
Yes	0.94	0.45 – 1.98	0.45 – 1.87

**Penicillin with Beta Lactamase Inhibitors**			
No	Reference		
Yes	1.15	0.74 – 1.78	0.73 – 1.82
**Elixhauser Score**			
Unit Increase	1.05	1.02 – 1.07	1.01 – 1.08
**Length of Index Stay (Day)**			
Unit Increase	0.98	0.96 – 1.00	0.96 – 1.00
